# Psychophysical stress and strain of maritime pilots in Germany. A cross-sectional study

**DOI:** 10.1371/journal.pone.0221269

**Published:** 2019-08-15

**Authors:** Filip Barbarewicz, Hans-Joachim Jensen, Volker Harth, Marcus Oldenburg

**Affiliations:** Institute for Occupational and Maritime Medicine (ZfAM) Hamburg; University Medical Center, Hamburg-Eppendorf, Germany; University o f Genoa, ITALY

## Abstract

**Introduction:**

Maritime pilots work in an irregular deployment system (rotation system) with unpredictable work assignments under high levels of physical and mental stress. Fatigue or chronic diseases, e.g. coronary heart disease, peptic ulcers or gastritis can occur as a consequence. This can lead to long-term limitations of pilots’ work ability. The aim of this study is to analyse current stress and strain in maritime pilots.

**Methods:**

Initially, all German pilots were interviewed with an online questionnaire about their living and working situation (response rate 43%). Subsequently, a medical and psychological examination of a random sample was carried out with pilots working in a 4-month rotation system compared with those working in a 1-week system. Most of the measurements took place at the beginning and the end of continuous work assignments each lasting several weeks (pre vs post-rotation). The questionnaires RESTQ-work 27, Resilience Scale RS-13 and Berlin Questionnaire were used as well as a sleeping diary. Furthermore, cardiovascular parameters (during rest and under ergometric stress), activity and blood parameters, urine stress hormones, and the pupillary unrest index were surveyed.

**Results:**

60 pilots were recorded with an average age of 48.7 years (SD 8.3 years). Among the parameters collected, there were no significant differences between pre and post-rotation examinations. Pilots with a 4-month rotation system experienced a much higher subjective strain level in RESTQ work-27 (OR 10.12 (95% CI 1.21–84.59)). According to the sleep diaries of the pilots working in a 4-month rotation system, reduced levels were found concerning the pre and post-rotation subjective performance level (p = 0.042 and 0.029), subjective sleep duration (p = 0.032) and current subjective feeling post-rotation (p = 0.036). Objectively measured arterial hypertension was significantly more frequent among pilots working 4 months at a time (OR 21.41 (95% CI 1.26–364.05)). In addition, elevated levels of total cholesterol, triglycerides and uric acid were more common among this group of pilots (p = 0.038, p = 0.033 and p = 0.038). In particular, the risk of hypertriglyceridemia was increased (OR 4.41 (95% CI 1.15–16.91)).

**Discussion:**

Maritime pilotage represents a very straining profession that has been studied very little up to this point. The present results indicate that 4-month rotation systems lead to higher levels of subjective and objective strain than 1-week rotation systems. Interventions are therefore recommended; especially a change in the rotation system should be considered.

## Introduction

According to the German Maritime Pilots Law, constant availability of pilots is required at any time of day or night, 365 days a year. This is monitored by regional supervisors so that all ships can always be served without delay. The irregular arrival of ships at pilots’ transfer stations makes it difficult to anticipate deployment requirements. The length of standby time depends on traffic and can therefore differ widely. Overall, the current deployment system requires a high degree of flexibility from pilots and their families, especially since there is a shift in generations and paradigms in pilotage. On the one hand, an increasingly higher average age of pilots as well as a simultaneously increasing employment rate among their spouses and life partners can be seen. On the other hand, a prioritisation of free time over work is observed [1].

Today, shipping without pilotage is unthinkable. Pilots are responsible for the safe navigation of ships through restricted and challenging waterways in controlled ports [2]. During their mostly very long work assignments, they are often isolated in a constantly changing work environment [3]. Pilots’ stress is unevenly distributed over their entire working life [4] and can lead to chronic fatigue. Acute fatigue is particularly pronounced after prolonged night shifts [5].

Further investigations point to a multitude of health-related influences in pilotage. In addition to sleep problems [6], these influences can lead to cognitive disorders, impaired alertness [7], a clinical manifestation of sleep apnea syndrome and to accidents [8]. Older studies from 1980 and 1990 have already demonstrated a higher risk for pilots to develop diseases of the cardiovascular system and the psyche [9] compared to comparative populations. According to these older studies, high levels of occupational strain in pilotage can chronically increase the risk of manifested disease, e.g. coronary heart disease [10–12], peptic ulcer or chronic gastritis [13]. There are several longitudinal studies with comparable findings [14].

Due to the unscheduled and unpredictable nature of their work assignments, pilots often also exhibit an unhealthy lifestyle (nicotine consumption, obesity, little exercise during leisure time) [15, 16]. A recent systematic review revealed an increase in triglyceridemia in workers exposed to prolonged stress [17]. The (physical and mental) health and the stress and strain level of this important professional group for world trade have so far been insufficiently investigated only in isolated older surveys [3]. In particular, there are gaps in literature about the influence of pilots’ working systems on strain. The aim of this study is to record and evaluate the current load and strain on pilots (for example, because of their rotation system or lack of sleep) during their on-board work assignments on an individual level.

## Methods

Rotation systems within the German pilots‘ associations ("rotation systems") differ considerably, depending on the district [1]. The most common systems last 1 week (2 associations) or 4 months (4 associations). Harbour pilots use a 1-week system in which pilots alternate between 8 days on and 6 days off work. Several cycles of work and free time are followed by three weeks off. The systems of sea and channel pilots’ associations in Germany are similar. Both associations predominantly use a 4-month system; a 4-month working period (2 or 4 free days per month) is followed by three to four weeks’ leave. It is assumed that there are differences between harbour and sea pilots. Whereas harbour pilots perform sophisticated manoeuvres in the harbour, sea pilots have a challenging vessel transfer in sea conditions.

### Sample collection

In order to estimate the willingness to participate, all 930 pilots in Germany were invited to take part in a voluntary online survey and 401 answered (participation rate of 43%). All 6 pilots’ associations using a 1-week or 4-month system were included in this study. A random sample of 17% was chosen from this pool of 368 pilots. To reduce confounders, frequency matching was performed by age groups, pilot‘s associations and partnership. As required for matching procedures, further statistical analysis has been adjusted by these variables. The time-consuming examinations (about 2 hours per pilot and examination) took place from May 2017 to March 2018 at the respective pilots’ stations.

Participation in this study was voluntary and participants provided a written consent in advance. The ethic committee of the Hamburg Medical Association, Germany, approved the study and gave a positive ethics vote (PV no. 5498).

### Investigation time

The examinations were carried out at the beginning of a work assignment (after holiday = pre-rotation) and at the end of the following work assignment lasting several weeks (before holiday = post-rotation) (Table 1). Stress (rotation system with appropriate working hours, pilots’ associations, operation report, number of steps, sleep duration) and resulting strain (biomonitoring, load ergometry, pupillometry, questionnaires) was recorded on an individual level, but examined on a group-based level (pre- and post).

**Table 1:** Examination time.

instrument	examination time		
	*pre-rotation*	*in the mid of rotation*	*post-rotation*
**operation report including heart rate and activity monitoring**		**x**	
**pupillometry**	**x**		**x**
**load ergometry**	**x**		
**blood analysis**			**x**
**stress hormones**	**x**		**x**
**questionnaires**	**x**		
**sleep diary**	**x**		**x**

### Operation report

Operation reports were used to record the following operation phases: stay at pilot station, cross over by transfer boat, way to bridge, pilotage on bridge, departure from ship, transfer to pilot station and rest time at pilot station. This investigation lasted 3 days (72 hours) to obtain a representative examination period of the normal pilot's work assignment and took place during standardized points of time (for 4-month ROS the examination time was 2 months +/- 2 weeks, for 1-week ROS the 3rd - 5th day +/- 1 day after ROS beginning).

Heart rate was measured continuously for the 3 days with the transmitter / receiver unit RS800CX Polar Electro in each of these exertion phases defined in the operation report. In addition, synchronous continuous activity was monitored using the Bodymedia SenseWear Pro 3 armband monitor. The monitor, which is worn on the right upper arm, analyses the profile of physical activity (movement, lying down, sleeping) [18, 19].

### Biomonitoring

Lipids (total cholesterol, LDL, HDL cholesterol, triglycerides), liver (ASAT, ALAT, yGT), metabolic (spontaneous glucose, HbA_1c_, uric acid) and renal values (creatinine, calculated GFR to CKD-EPI) in the blood were analysed for the screening of (cardiovascular) risk factors. According to the “Labor Lademannbogen Hamburg” laboratory, the following values were defined as the upper limit of the respective reference range: 200 mg/dl for cholesterol, 150 mg/dl for triglycerides, and 7 mg/dl for uric acid in men. In addition, the stress hormones epinephrine, norepinephrine and dopamine in 24-h urine were determined. Elevated stress hormones were defined as epinephrine > 20 μg/d, norepinephrine > 80 μg/d or dopamine > 460 μg/d.

The limit for arterial hypertension was defined as elevated blood pressure (systolic >140 mmHg or diastolic >90 mmHg) [20].

### Load ergometry Chester step test

The Chester step test is a multi-step submaximal test for determining age-related endurance capacity [21, 22] with a very high correlation to the medical gold standard of spiroergometry [23]. It determines individual oxygen consumption (VO_2_) [24, 25].

### Pupillometry

The Pupillographic Sleepiness Test is an objective method for recording daytime sleepiness by recording spontaneous and unconscious pupillary oscillations without light stimulus. With pupillometry, using the AmTech Fit-For-Duty measuring device, pupil width was continuously recorded for 11 minutes by a camera integrated in light-shielding glasses and pupil oscillations evaluated. The result is a pupillogram which can be used to deduct the pupillary unrest index (rPUI), a parameter for the variance of the pupil’s diameter. Based on a reference collective, results were categorically interpreted as normal (rPUI ≤ 1.02), marginal 1.02 < rPUI ≤1.53) or pathologic (>1.53) [26, 27]. An increase in daytime sleepiness was defined as deterioration by at least one category level. The Pupillographic Sleepiness Test is a reliable measurement to assess sleepiness [28–30].

### Questionnaires

RESTQ-work 27 was used to record strain and recovery at work [31]. The Berlin Questionnaire was applied to determine the risk of obstructive sleep apnea syndrome (OSAS) [32]. Following the recommendations of the German Society for Sleep Research and Sleep Medicine, the validated short version of the "evening morning protocols" was also used to record individual pathologies of sleep (sleep diary) [33, 34]. In addition, Resilience Scale (RS-13) captured psychological resilience as a permanent individual resource [35]. The RS-13 is an economic measuring instrument for measuring resilience as trait stability. Resilience is seen as a construct that encompasses constitutional personality traits and coping skills [35]. The RS-13 therefore is not sensitive to change over time.

### Statistics

Statistical analysis was performed with SPSS (version 24, IBM Corperation). Parametric (Student's T, Chi Square, Fisher Exact) and non-parametric tests (Mann Whitney U, Friedman) were used in addition to descriptive statistics (mean with standard deviation (SD)). P-values lower than 0.05 marked error probability. First, a separate comparison of stress and strain parameters was performed between the time of examination (pre and post-rotation). Subsequently, the rotation systems (ROS) were compared. Crude odds ratio (OR) and adjusted OR (adjusted for pilots’ associations and age) including 95% confidence intervals were calculated by binary logistic regression.

## Results

### Demographic data

Demographic data (age, weight, BMI, partnership, children) of 1-week ROS (n = 12) and 4-month ROS (n = 48) showed no significant differences partly as a consequence of matching (Table 2). Nevertheless, more obese pilots (BMI ≥ 25) were found in the 4-month ROS. In addition, the latter pilots tended to rarely have a (working) partner or children. The results of RS-13 showed no significant differences, although 4-month ROS pilots often had lower resilience than their colleagues.

**Table 2:** Demographic characteristics.

	1-week ROS	4-month ROS	p
	(n = 12)	(n = 48)	
**age,** years (SD)	50.0 (6.7)	48.3 (8.6)	0.536^1^
**weight,** kg (SD)	88.5 (11.4)	95.4 (16.1)	0.318^2^
**BMI**, kg/m^2^ (SD)	26.4 (3.6)	28.6 (3.9)	0.070^1^
≥ 25, n (%)	7 (58.3%)	40 (83.3%)	0.431^3^
**partnership,** n (%)	12 (100%)	39 (81.3%)	0.214^3^
working partner, n (%)	11 (91.7%)	24 (50.0%)	0.079^4^
working hours/week (SD)	22.6 (11.3)	14.5 (16.4)	0.102^2^
**children,** n (%)	12 (100%)	36 (75.0%)	0.153^3^

^1^Student's T test

^2^ Mann Whitney U test

^3^Chi Square test

^4^ Fisher Exact test

### Questionnaire and biometric data

None of the parameters showed a significant difference between pre and post-rotation examination (Table 3). Nevertheless, a trend emerged: In their post-rotation sleep diaries, pilots stated to subjectively have a little less strength (lower performance) and to be more tense (current feeling). Sleep efficiency also tended to be worse during post-rotation evaluation. Generally, reduced activity (fewer steps, longer sleeping time and stay in bed) was recorded post rotation. Daytime sleepiness (rPUI) was higher post rotation, as expected. In addition, the stress hormones epinephrine and dopamine in urine tended to be higher post rotation.

**Table 3:** Questionnaire and biometric data.

	pre-rotation	post-rotation	p
**sleeping diary**			
performance (SD)	2.4 (0.7)	2.1 (0.8)	0.210^1^
sleep during the day, min (SD)	42.8 (32.3)	41.4 (61.7)	0.894^1^
sleep at night, h (SD)	6.1 (2.9)	6.8 (4.6)	0.687^1^
feelings: tension (SD)	4.1 (0.8)	4.4 (1.2)	0.097^1^
total wearing time of activity monitor, h (SD)	66.6 (26.1)	69.2 (44.0)	0.803^1^
**activity data**			
steps/day (SD)	9,730 (4,096)	9,337 (5,147)	0.663^1^
physical activity, h/day (SD)	2.1 (1.2)	2.0 (1.2)	0.401^1^
sleep time, h/day (SD)	6.1 (2.9)	6.8 (4.6)	0.687^1^
time in bed, h/day (SD)	7.7 (3.4)	8.7 (5.5)	0.619^1^
sleep efficiency, % (SD)	78.3 (11.3)	71.7 (10.8)	0.233^1^
energy consumption, kJ/day (SD)	13,435 (4,480)	13,504 (6,087)	0.589^1^
**pupillometry**			
rPUI (SD)	0.9 (0.4)	1.0 (0.5)	0.138^1^
**heart rate**, bpm (SD)	75.9 (8.5)	75.6 (7.0)	0.662^1^
**blood pressure**			
systolic, mmHg (SD)	136.4 (11.6)	135.9 (11.3)	0.631^2^
diastolic, mmHg (SD)	85.1 (8.5)	84.6 (7.2)	0.403^2^
**24-h-urine sampling**			
norepinephrine, μg/day (SD)	47.2 (26.7)	49.0 (19.3)	0.841^1^
epinephrine, μg/day (SD)	6.0 (4.5)	6.7 (3.9)	0.724^1^
dopamine, μg/day (SD)	273.1 (147.7)	298.2 (149.1)	0.348^1^

^1^Student's T test

^2^ Friedman test

Due to a lack of significant differences, data from examination times (pre and post-rotation) was summarised and the rotation systems were compared. Significant differences were detected: in RESTQ work-27, pilots of the 4-month ROS rated the subjective strain as 10 times higher (Table 4). Among 4-month ROS pilots, arterial hypertonia was significantly more frequent (OR 21.41 (95% CI 1.26–364.05)). Furthermore, elevated blood values more frequently appeared concerning total cholesterol (224.7 mg/dl vs 199.4 mg/dl, p = 0.038), triglycerides (220.9 mg/dl vs 148.5 mg/dl, p = 0.033) and uric acid (6.1 mg/dl vs 5.6 mg/dl, p = 0.038). In particular, the risk of hypertriglyceridemia was increased (OR 4.41 (95% CI 1.15–16.91)). Altogether, the PROCAM score did not differ between both rotation systems (33.4 points vs 33.3 points). Further blood and urine parameters (stress hormones) showed no significant differences between both systems.

**Table 4:** Questionnaire and biometric data (pre and post-rotation data summarised).

	1-week ROS	4- month ROS	crude OR (95% CI)	adjusted OR^*#*^ (95% CI)
samples, n	24	96		
**RESTQ-work 27,** n (%)				
elevated level of strain*	**1 (8.3%)**	**23 (47.9%)**	**10.12 (1.21–84.59)**	**9.14 (1.10–83.54)**
**Berlin Questionnaire,** n (%)				
elevated OSAS risk*	2 (16.7%)	13 (27.1%)	1.86 (0.36–9.63)	1.43 (0.27–7.61)
**activity data**, n (%)				
steps/day <10,000	10 (58.8%)^1^	30 (71.4%)^2^	1.75 (0.54–5.67)	1.63 (0.50–5.31)
energy consumption >median^3^	9 (52.9%)	18 (42.9%)	0.77 (0.26–2.27)	0.60 (0.20–1.82)
sleep efficiency <80%	8 (47.1%)	20 (47.6%)	1.02 (0.33–3.16)	1.01 (0.18–2.30)
**heart rate,** n (%)				
median^4^	2 (8.3%)	12 (12.5%)	1.57 (0.33–7.54)	1.31 (0.27–6.42)
**arterial hypertension**, n (%)	**0 (0.0%)**	**29 (60.4%)**	**21.41 (1.26–364.05)**	**17.31 (1.15–85.27)**
ergometry*^5^, n (%)				
very good	2 (16.7%)	19 (39.6%)	3.28 (0.65–16.63)	2.93 (0.57–14.99)
good	8 (66.7%)	23 (47.9%)	0.30 (0.06–1.60)	0.11 (0.01–1.28)
at least average	2 (16.7%)	6 (10.4%)	0.26 (0.03–2.36)	0.08 (0.02–3.66)
blood analysis*^&^, n (%)				
hypercholesterolemia	6 (50.0%)	33 (68.8%)	2.20 (0.61–7.96)	2.39 (0.54–10.71)
hypertriglyceridemia	**4 (33.3%)**	**33 (68.8%)**	**4.41 (1.15–16.91)**	**5.51 (1.10–27.68)**
hyperglycemia	1 (8.3%)	5 (10.4%)	1.28 (0.14–12.10)	0.78 (0.08–7.39)
hyperuricemia	0 (0.0%)	11 (22.9%)	7.67 (0.42–139.74)	3.63 (0.20–65.28)
**24-h-urine sampling**^&^, n (%)				
elevated stress hormones	4 (16.7%)	6 (6.3%)	0.33 (0.09–1.29)	0.72 (0.21–2.45)
**pupillometry**, n (%)				
normal	18 (75.0%)	62 (64.6%)	0.61 (0.15–2.6)	0.59 (0.21–1.62)
marginal	3 (12.5%)	26 (27.1%)	3.55 (0.85–14.74)	2.60 (0.72–9.45)
pathological	3 (12.5%)	8 (8.3%)	0.60 (0.13–2.72)	0.64 (0.16–2.61)

Significant differences in bold

^#^adjusted for pilots’ associations and age

*only pre-rotation evaluation (12 vs 48 pilots)

^&^reference values see Methods

^1^ data incomplete (n = 17)

^2^ data incomplete (n = 42)

^3^ median 13,154 kJ/day

^4^ median 76 bpm

^5^ age-related according to test protocol

No significant differences were found in subjective assessment according to sleep diaries or in activity data (steps, physical activity, sleep duration and energy consumption). In ergometry, the majority of participants showed good to very good results with an average VO_2_ of 44.4 ml O_2_/kg/min without significant differences between ROS. Furthermore, no differences in daytime sleepiness defined by rPUI >1,02 were noted. Here, 4-month ROS pilots tend to be more tired than their counterparts (rPUI pre-rotation 0.88 vs 0.80 and rPUI post-rotation 0.98 vs 0.89) and more of them experienced an increase in daytime sleepiness between pre and post-rotation (deterioration by one category: 20.8% vs 16.7%). The Berlin Questionnaire found evidence of obstructive sleep apnea syndrome in 15 pilots (25%). Here, more OSAS risk cases were recorded among 4-month ROS pilots, without reaching a significant level. An adjusted OR was calculated in order to detect population-specific and age-specific differences in stress, whereby no statistically significant influence of district and age could be determined for any of the investigated parameters.

The different operation phases during a typical pilotage work assignment (e.g. boat transfer), presented in methods, did not show any significant differences in heart rate and activity data, neither intraindividually nor between both systems.

Further differentiation of pre and post-rotation data revealed the following significant differences between both ROS (4-month ROS vs 1-week ROS) in contrast to the overall data in Tab. 2: Subjective performance according to sleep diary (pre-rotation: 2.5 vs 2.0, p = 0.042; and post-rotation: 2.3 vs 1.7, p = 0.029), sleep duration during day (post-rotation: 36.8 min vs 62.7 min, p = 0.032) and current subjective tension (post-rotation: 4.2 vs 5.0, p = 0.036).

## Discussion

Maritime pilots’ occupational health has so far scarcely been studied, and the existing older studies about this professional group suggest high levels of psychophysical stress and strain, especially an increased risk of cardiovascular diseases. However, compared to a comparative German population, pilots can be attributed a similar (low) cardiovascular risk (4.9% vs 2.9% according to prospective cardiovascular Munster (PROCAM) study score [36–39]. This confirms the results of an earlier study on the health status of Scottish pilots between 1988 and 2012, indicating that the number of cardiovascular risk factors of pilots has decreased compared to older studies (also as an effect of described lower nicotine and alcohol consumption) [17]. In this study, ergometry even showed above-average cardiovascular performance [22]. The currently estimated relative high resilience of the pilots (mean points 74 vs 70 in comparable population) [33] matches the results in ergometry.

In pupillometry, more indications of sleepiness were found among maritime pilots than in the general population ashore (30% vs 13%) [26, 27], but similar results compared to other occupational sectors with elevated levels of fatigue (lorry drivers, bus drivers and shift workers: 30%-35%) [40, 41]. The assessed risk of obstructive sleep apnea syndrome was also higher than in the general population (25% vs 10%) [42], but comparable with the risk of lorry drivers (30%) [40, 43, 44]. Overall, compared to appropriate reference groups, it appears that pilots have a similarly high fatigue-related health risk [45]. Therefore, countermeasures, such as training in anti-fatigue management, are recommended.

In an earlier study in 1984, a presumably stress-induced increase in heart rate during pilots’ transfer from pilot boats to ships was reported [46]. This could not be confirmed in this study. A possible explanation irrespective of the ROS is e.g. the currently facilitated transfer manoeuvre compared to the work situation in 1980 (through better and more stable transfer boats) or an optimised preparation for upcoming duty with innovative information technology, which provides information both on the ship and current weather conditions.

In the comparison of pre and post-rotation data, no significant work-related strain was observed in this study, although there was a trend. This trend indicates that pilots have shown signs of fatigue in the course of their rotation: for example, post rotation: a slight decrease in subjective performance and an increase in tension were recorded − corresponding to an objectively measurable slight decrease in sleep efficiency. In addition, it was noted that 4-month ROS pilots tended to be more obese, to have fewer relationships and fewer children. This trend could indicate that a constant 4-month ROS interferes with relationships and family planning.

There were significant differences in occupational strain in the comparison between both ROS. The average energy consumption per day of more than 13,000 kJ corresponds to a higher energy turnover (about 9,260 kJ/day in a moderately active 40-year-old person) [47]. Average sleep duration of about 6.5 hours per day is considered to be low (6–8 hours per day in a comparable general male population) [48]. The calculation of adjusted OR did not reveal any district-specific or age-dependent differences. This suggests that the recorded stress and strain were representative and independent of age.

Due to longer work assignments in one stretch 4-month ROS seem to be measurably higher in stress, leading to an increased subjective strain (RESTQ-work 27) and worse subjective assessments in sleep diaries (pre and post-rotation performance, post-rotation sleep duration during the day and current tension post rotation). In accordance with these findings, a higher rate of arterial hypertension and increased blood lipid levels were detectable among 4-month ROS pilots. Duration of work and restriction of leisure time planning, as reported in interviews, may lead to an unhealthy lifestyle with mental compensatory behaviour ("stress eating") and may be responsible for the poorer biometric data of these pilots [1].

The previously conducted studies on the stress level of pilots have already described difficult accessibility or little willingness to participate which have resulted in small study samples (mean n = 74, range 1–434) as in this study [3]. Due to the low number of subjects in this examination, bias cannot be ruled out (uncertainty as to the representativeness of this study population due to the low participation rate of volunteers) as a limitation of this study. In addition, when interpreting the results, a possible "healthy worker effect" should be taken into account which could lead to an underestimation of stress and strain levels in maritime pilotage.

Furthermore limitations of the instruments used have to be taken into account: The wearable device does not offer information about sleep architecture. It is more suitable for measuring bed rest [49]. Additionally, the device may underestimate sleep efficiency because of imprecise recording of lying time. Pupillometry is not yet reliable as a screening test for sleepiness. A further analysis of sleep can only be evaluated by polysomnography in sleep laboratories [50].There are still interesting aspects for further research and statistical analysis (e.g. examination within groups across the time periods declared in the operation report to record subtle changes (e.g., night vs. day, day to day etc.) and to determine differences in the type of pilots’ work. Nevertheless, this unique study describes current maritime pilots’ stress and strain using a variety of reliable and elaborate methods and analysing the influence of their rotation system on strain.

## Conclusions

Duration and intensity of the multiweek work intervals seem to have a significant impact on the pilots’ strain: pilots working in 4-month ROS have a higher strain level than pilots in a 1-week ROS. It seems necessary to change the rotation system towards a shorter and therefore better predictable and more family-compatible work system. An already occurring paradigm change in pilotage is to be included in long-term planning. These interventions should be reviewed in the future through follow-up studies and continuously optimised as necessary.
